# Supplementary material for: Design and Synthesis of a Novel NIR Celecoxib-Based Fluorescent Probe for Cyclooxygenase-2 Targeted Bioimaging in Tumor Cells
Source: Molecules. 2020 Sep 4;25(18):4037. doi: 10.3390/molecules25184037 (PMC7570625; doi:10.3390/molecules25184037)
Supplement: Supplementary file 1 [file molecules-25-04037-s001.pdf]

Supplementary Material

Design and Synthesis of a Novel NIR Celecoxib-Based Fluorescent Probe for Cyclooxygenase-2 Targeted Bioimaging in Tumor Cells

Xinli Wang,[1,2] Liye Wang[2], Lijun Xie,[2,3] Zuoxu Xie,[2] Li Li,[2] Dinh Bui,[2] Taijun Yin,[2] Song Gao,[2] and Ming Hu[2,] *

[1]Department of Medical Oncology, Fujian Medical University Union Hospital, Fuzhou, Fujian 350001, PR China

[2]Department of Pharmacological and Pharmaceutical Sciences, College of Pharmacy, University of Houston, Houston, TX 77204

[3]Fujian Provincial Key Laboratory of Screening for Novel Microbial Products, Fujian Institute of Microbiology, Fuzhou, Fujian 350007, PR China

MS of Compound 2

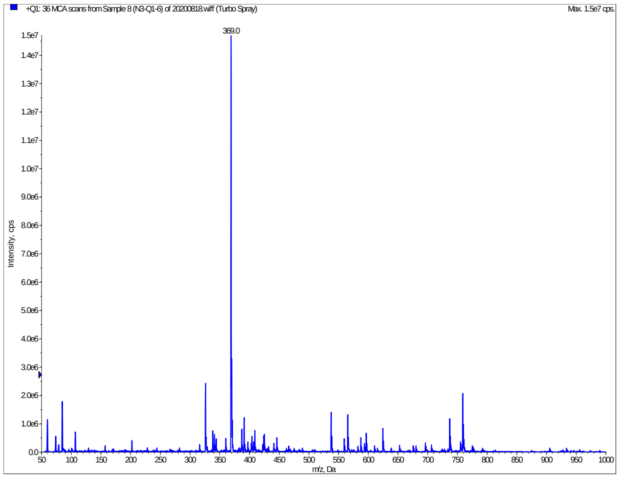

^{1}H-NMR of compound 2

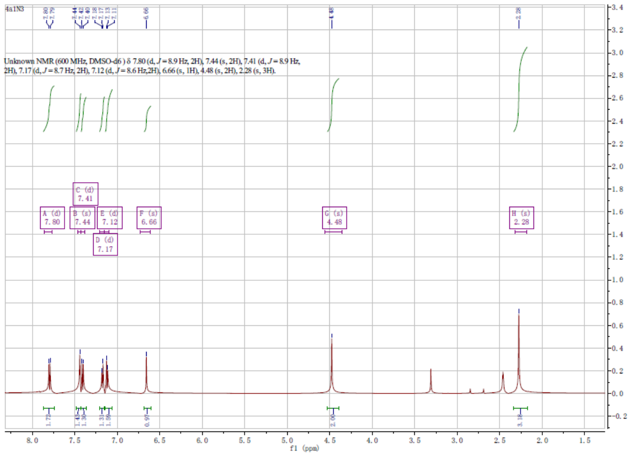

^{13}C-NMR of compound 2

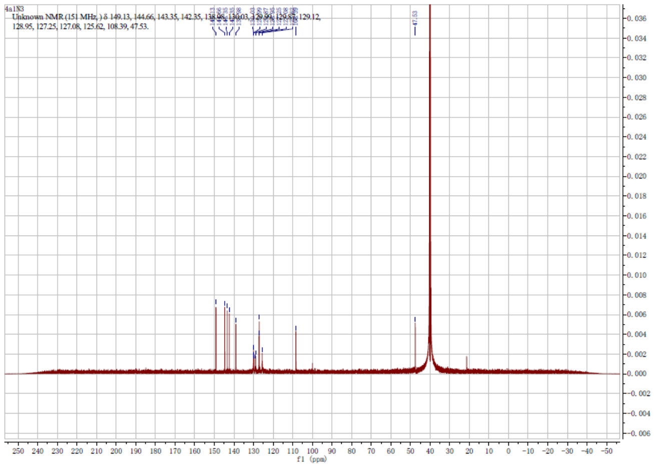

MS of Compound 3

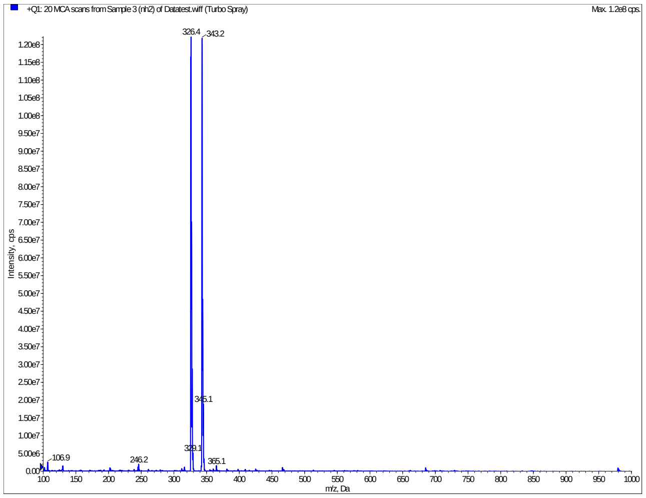

^{1}H-NMR of compound 3

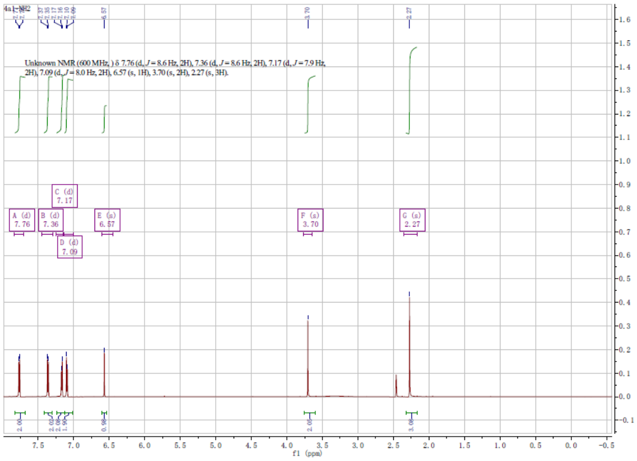

^{13}C-NMR of compound 3

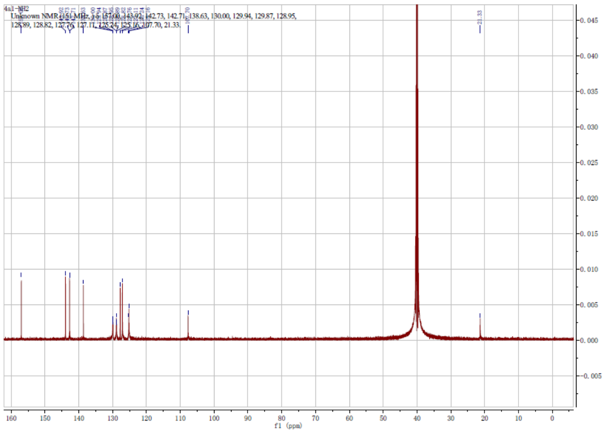

MS of Compound CCY-5

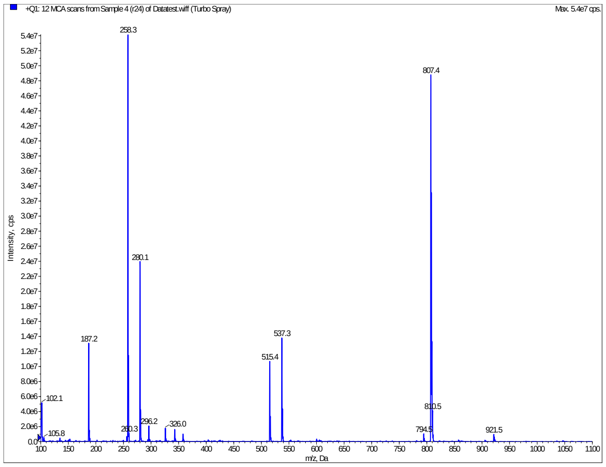

^{1}H-NMR of CCY-5

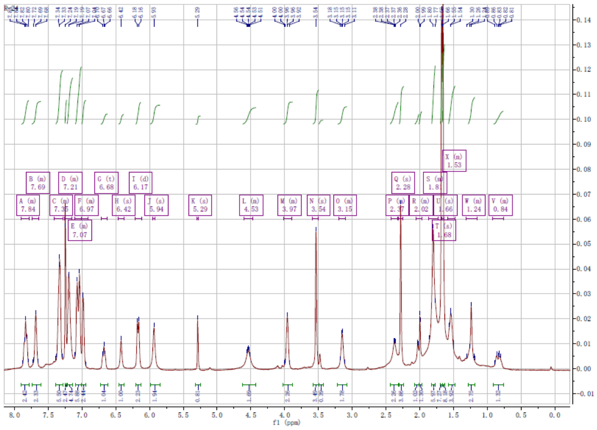

^{13}C-NMR of CCY-5

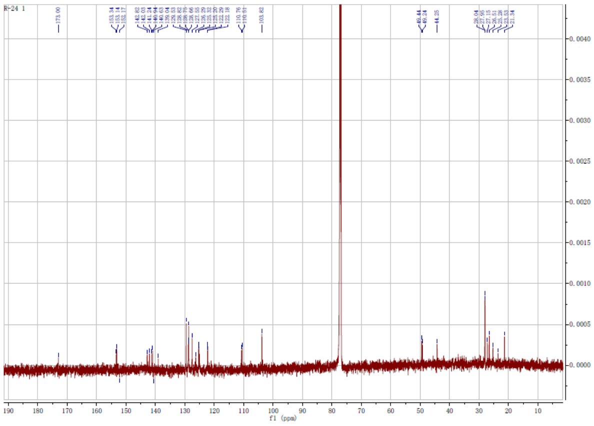

Fluorescent microscopy images of normal and cancer cell lines

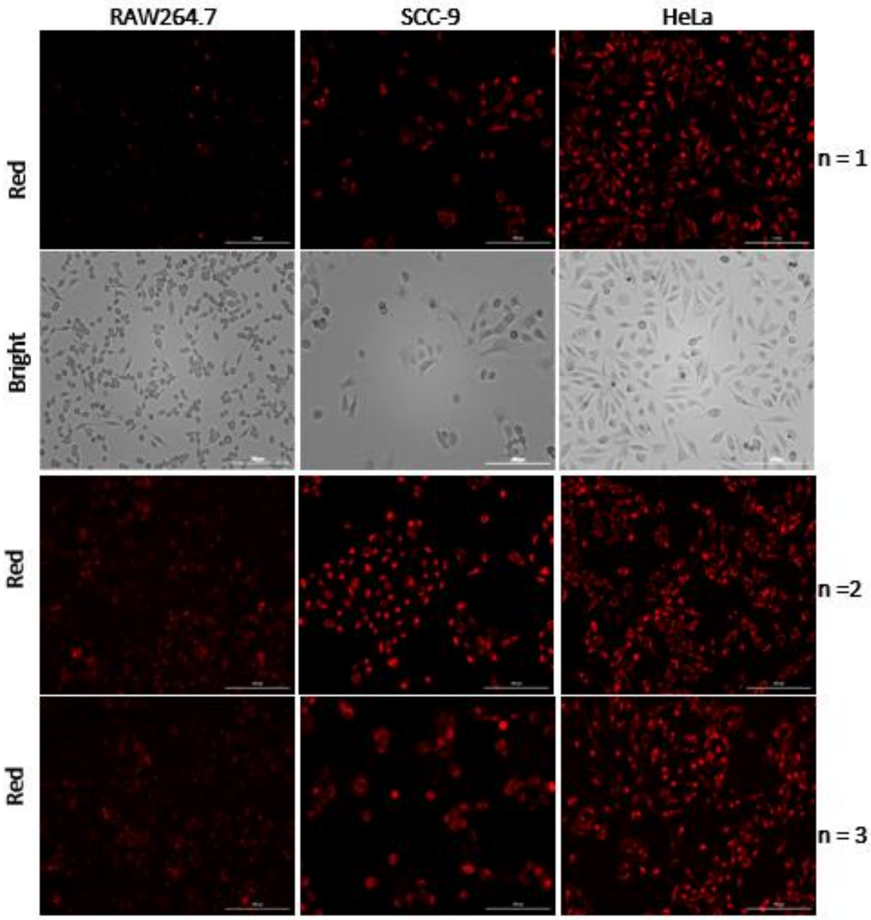

Fig S1. Fluorescence images of RAW264.7, SCC-9 and HeLa cell lines with fluorescent microscopy. All cells were treated with CCY-5 (1 μM) for 30 mins. (λex: 628 nm; Red, λem: 685 nm, All the images share the same scale bar: 200 μm).

Fluorescence images of LPS-treated cells

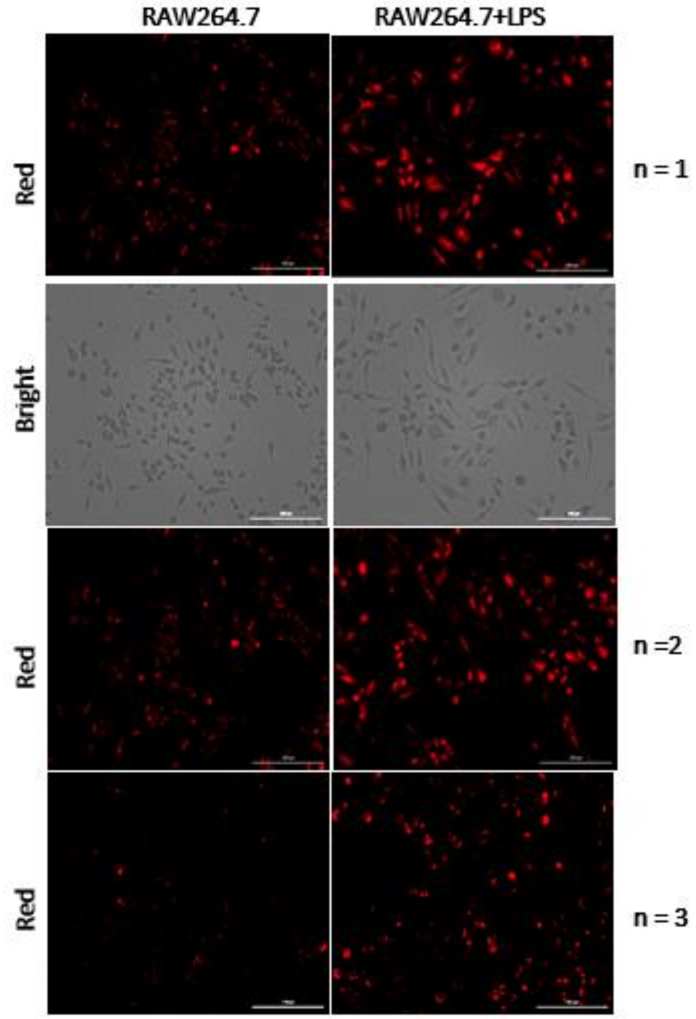

Fig S2 Fluorescent images of RAW264.7 and inflamed RAW264.7 cells. Fluorescence images of LPS-induced inflamed cell lines (RAW264.7 cells). Cells were incubated with LPS (1 ug/mL) for 12 h, followed by CCY-5 treatment (1 μM) for 2 h (λex: 628 nm; Red, λem: 685 nm, All the images share the same scale bar: 200 μm).

COX-2 inhibitory effect

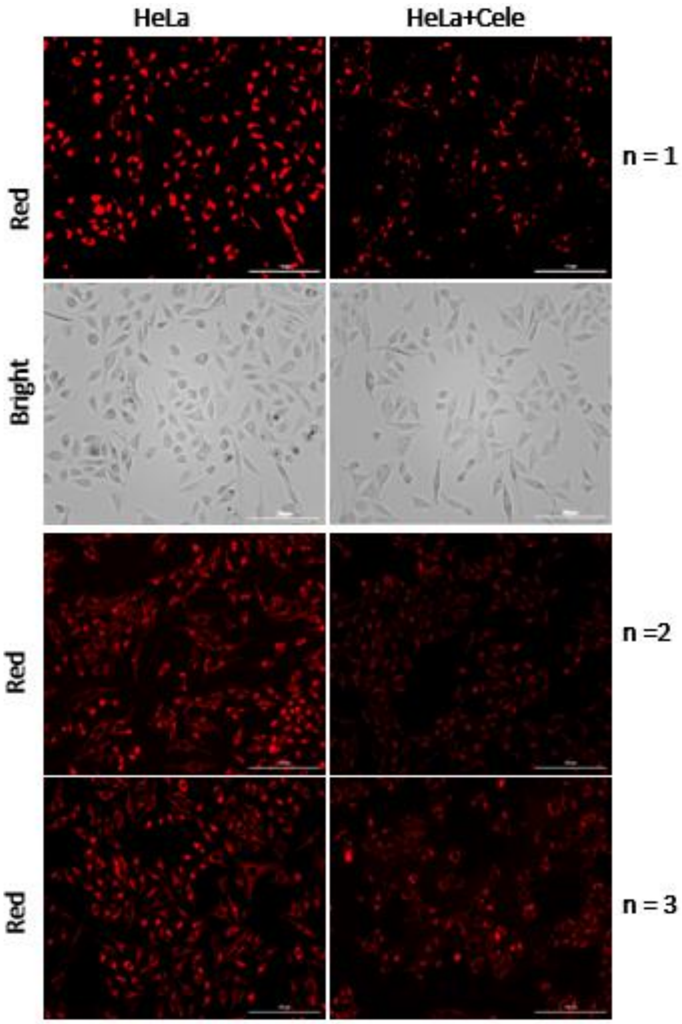

.Fig S3. COX-2 inhibitory effect. Fluorescent images of HeLa and HeLa cells pre-treated with COX-2 inhibitors (Cele = Celecoxib), fluorescent microscopy images with treatment of CCY-5 (1 μM) for 30 mins (λex: 628 nm; Red, λem: 685 nm, All the images share the same scale bar: 200 μm).
